# Fast preparation of carbon spheres from enzymatic hydrolysis lignin: Effects of hydrothermal carbonization conditions

**DOI:** 10.1038/s41598-018-27777-4

**Published:** 2018-06-22

**Authors:** Haiyan Mao, Xianwen Chen, Runzhou Huang, Minzhi Chen, Rui Yang, Pin Lan, Meijun Zhou, Feng Zhang, Yu Yang, Xiaoyan Zhou

**Affiliations:** 1 College of Materials Science and Engineering, Nanjing Forestry University, Nanjing, 210037 China; 2 Department of Chemical and Biomolecular Engineering, University of California, Berkeley, CA 94720 USA; 3 Jiangsu Chenguang Coating Co., Ltd., Changzhou, 213164 China

## Abstract

This study explores the effect of carbon sphere preparation conditions on the morphology of the carbon spheres and the micropore development by fast potassium hydroxide activation via microwave heating. Enzymatic hydrolysis lignin is used as the precursor for carbon sphere preparation via environmentally friendly hydrothermal carbonization. The effects of various carbonization temperatures, carbonization times and reaction concentrations on the physical morphology of the carbon sphere surfaces are investigated. The Brunauer-Emmett-Teller surface area, yield and scanning electron microscopic images are used to characterize the carbon spheres. High carbonization temperatures and times result in large particle sizes, high sphericity, uniform size, and high dispersity of the carbon spheres. The best carbon spheres are obtained at 270 °C for 7 hours with a reaction concentration of 0.06 g ml^−1^ and a particle size of 3–6 μm. After activation, the Brunauer-Emmett-Teller surface area of the activated carbon spheres increases from 248 m^2^ g^−1^ to 1278 m^2^ g^−1^. Carbon spheres activated by treatment with fast potassium hydroxide and microwave heating can develop micropores that enhance the adsorptive capacity for small molecules, such as gases. Enzymatic hydrolysis lignin-derived carbon spheres formed via hydrothermal carbonization should be potentially sustainable materials applicable in energy and environmental fields.

## Introduction

Carbon spheres (CSs) have garnered significant interest among researchers because of their potential applications, e.g., in gas separation and as molecular sieves^1,2^, photonic band gap crystals^3,4^, catalyst supports^5,6^ and electrode materials for lithium ion batteries^7,8^. CSs serve as supporting substrates owing to their high surface area and high structural stability, among other properties. Compared with coal, petroleum, or their derived products, CSs derived from sustainable, inexpensive and environmentally benign biomass for energy storage have become particularly fascinating^9–14^.

Lignin is the most abundant aromatic polymer on earth and must be removed before natural biomass is converted to prepared biofuel^15^. However, in the preparation of biofuel ethanol using lignocellulosic materials, large quantities of enzymatic hydrolysis residues, whose main components are lignin, are produced. Research shows that 6–7 tons of cornstalk can produce 1 ton of fuel ethanol. That mass of cornstalk can also produce 1 ton of residue at the same time, which contains a large amount of (approximately 40~50%) enzymatic hydrolysis lignin (EHL), in addition to a small amount of non-hydrolyzed cornstalk and other impurities^15–17^. With the development of the lignocellulosic ethanol industry, EHL will inevitably become an abundant renewable resource. The value-added utilization of EHL can significantly reduce the production cost of lignocellulosic ethanol and effectively enhance the economic viability of the lignocellulosic ethanol industry. Unlike lignin made from traditional sulfite or alkaline pulping, EHL does not undergo alkaline or sulfite cooking and therefore better retains active groups containing more lignin, such as phenol hydroxyl and alcoholic hydroxyl. In addition, EHL exhibits small dispersivity and a low relative molecular weight relative to traditional industrial lignin and thereby enjoys broad application prospects. As the molecular chain of lignin contains a large number of phenyl rings, with a carbon content of over 50%, lignin is considered a suitable raw material for carbon materials^18,19^. However, EHL has not been further developed and utilized. In reported studies, EHL is only burned off as fuel and has a low added value, causing a serious waste of resources^20^.

Carbon materials can be prepared or manufactured from various types of raw materials whose innate characteristics can affect the properties and qualities of the resulting carbon^21^. Among the available techniques^22^, so-called hydrothermal carbonization (HTC) affords carbon spheres with superior and promising properties for various applications^23^. The HTC process is more environmentally friendly than other methods because it proceeds under a closed system and at low temperatures^24^. Mi *et al*. used a hydrothermal route in a stainless-steel autoclave with optimal reaction conditions of 500 °C and 12 h to successfully synthesize carbon microspheres with a regular shape and a diameter of 1 to 2 μm^25^. Simsir *et al*. investigated the hydrothermal carbonization of glucose, cellulose, chitin, chitosan and wood chips at 200 °C at processing times between 6 and 48 h. The average sizes of carbon spheres using glucose and chitosan were found to be 560 and 42 nm, respectively^26^. In 2009, Sevilla and Fuertes used glucose, sucrose and starch as raw materials to study the effect of hydrothermal carbonization on the structure and properties of carbon microspheres^27^. The researchers ultimately synthesized uniform spherical microparticles with a diameter of 0.4–6 μm and found that the microspheres exhibited a core-shell structure composed of a highly aromatic nucleus (hydrophobic) and a hydrophilic shell. The structures contained a high concentration of reactive oxygen functional groups (i.e., hydroxyl/phenolic, carbonyl, or carboxylic) and a large number of surface hydroxyl, carbonyl, keto, carboxyl groups. In addition, the results showed that the main factors affecting the size of the carbon microspheres were the concentration of the saccharide solution, reaction time and temperature. Finally, the size distribution of carbon microspheres formed at a high reaction temperature are highly uniform^27^. Despite the aforementioned efforts, to date, systematic investigations of the effects of various factors on the preparation of uniform and separate carbon spheres derived from EHL have not been widely reported. Thus, the development of a synthesis method using enzymatic hydrolysis lignin is well suited for this application. As commonly observed for HTC products, hydrothermal carbon spheres have no relevant porosity, which hinders their potential application for gas adsorption and energy storage^27,28^.

As discussed above, the use of EHL for preparing functional carbon materials via the HTC method is relatively simple. However, the processes and reactions involved in the HTC are highly complex. The surface of the CSs thus obtained consisted of abundant oxygen-containing functional groups, and the size and dispersion of the CSs could be modified by adjusting the reaction conditions. To gain separate, stable and uniform carbon spheres, this study used the EHL as the raw material and the HTC method to synthesize carbon spheres. The influence of reaction conditions on the morphology of carbon spheres was examined. The research involved the following: (1) use of the HTC method to investigate the effects of the EHL concentration, reaction temperatures and reaction times on the characterization of the microstructure and surface functional groups of the spheres via scanning electron microscopy (SEM) and Fourier transform infrared spectroscopy (FTIR) and (2) systematic examination of the effects of KOH and microwave heating on the porous structure and surface chemistry of carbon spheres. The derived CSs can be widely applied in hydrogen storage, capacitors and other applications. CSs have promising application prospects and offer many challenges for future study. Enzymatic hydrolysis lignin-derived carbon spheres activated by microwave heating and KOH should be potentially sustainable materials for application in environmental gas adsorption.

## Results and Discussion

### The effects of HTC conditions

#### Effect of reaction temperature on the morphology of CSs

**Figure 1:**
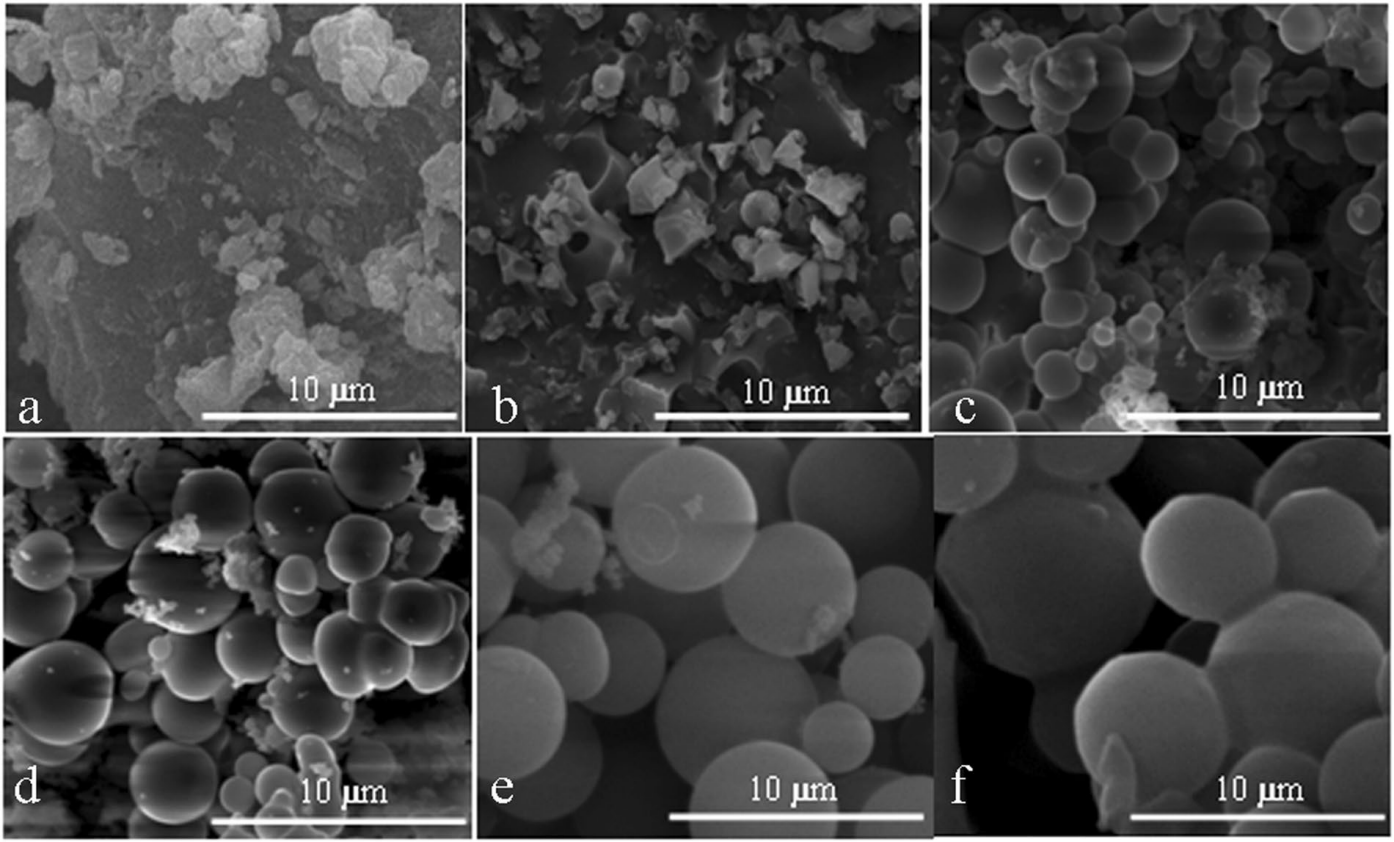
SEM images of extracted EHL (**a**) and carbon spheres formed at 220 °C (**b**), 230 °C (**c**), 250 °C (**d**), 270 °C (**e**), and 290 °C (**f**) (the carbonization time and concentration were 7 hours and 0.06 g ml^-1^, respectively).

Figure 1 shows SEM images of EHL-derived carbon spheres synthesized by HTC at different reaction temperatures. The figure shows that EHL was used as a raw material to synthesize CSs, and the carbonization time and concentration were 7 h and 0.06 g ml^−1^, respectively. CSs synthesized at different reaction temperatures showed variations in morphology, particle size and dispersion. At 220 °C, the prepared solid product (Fig. 1b) is quite similar to the original EHL in morphology (Fig. 1a), and no spherical structure appears. At reaction temperatures above 230 °C, CSs with a particle size of approximately 2 μm appears. Under these conditions, the surface of the CS was rather smooth. However, the size of the particles was not sufficiently uniform.

With the increase in the HTC reaction temperature (230–270 °C), the particle size of the derived CSs increased from approximately 2 to 3 μm (Fig. 1c,d), and the dispersion became slightly better. This finding indicates that increasing the reaction temperature is conducive to the hydrolysis of EHL and plays a decisive role in the formation of CSs. Moreover, the increase affects the particle size of CSs^29^. However, when the reaction temperature was increased from 270 to 290 °C (Fig. 1), aggregation and cross-linking of the CSs began to occur. Therefore, 270 °C is the optimal reaction temperature for CS synthesis.

#### Effect of reaction time on the morphology of CSs

**Figure 2:**
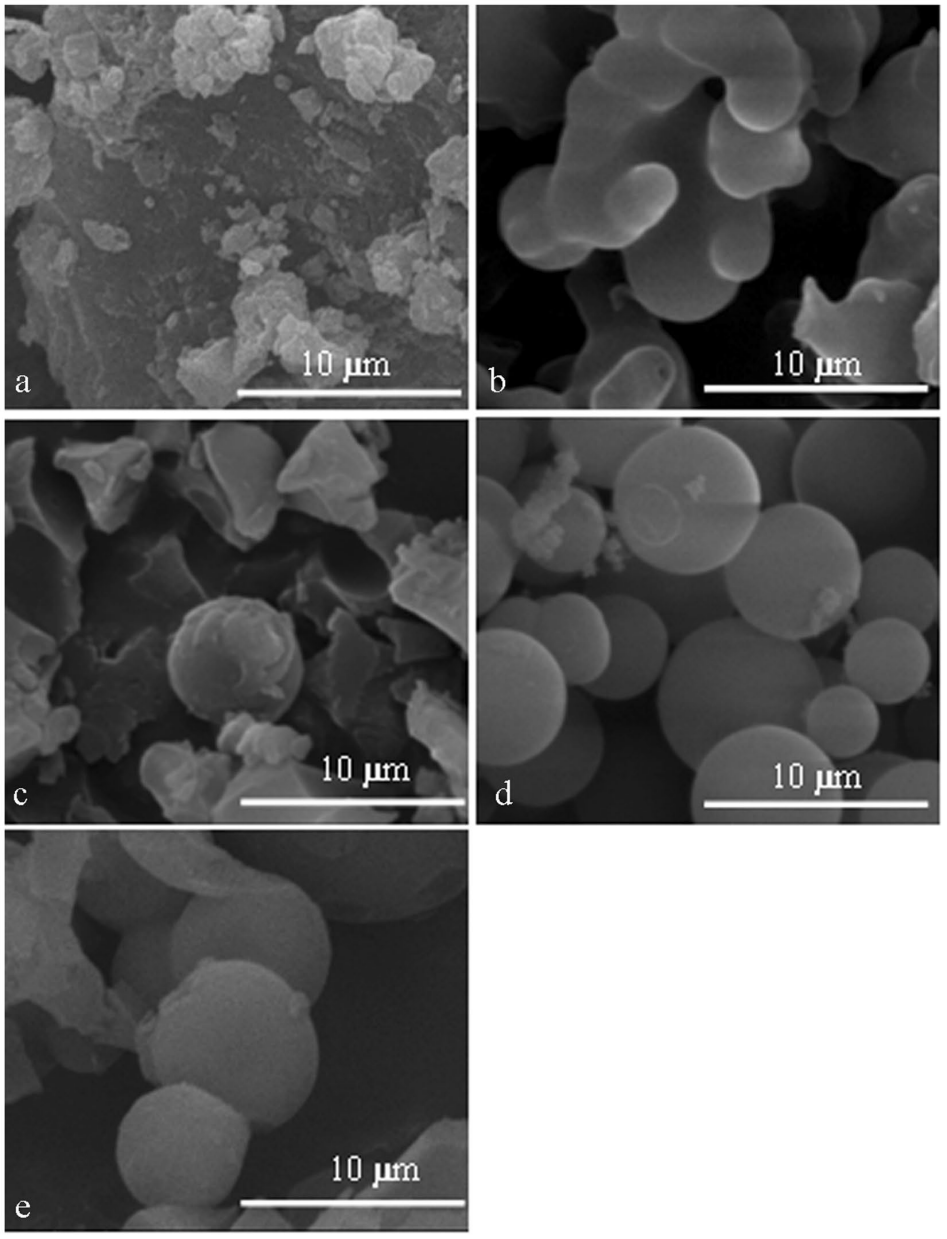
SEM images of extracted EHL (**a**) and carbon spheres formed at carbonization times of 3 h (**b**), 5 h (**c**), 7 h (**d**) and 9 h (**e**) (the carbonization temperature and concentration were 270 °C and 0.06 g ml^−1^, respectively).

Figure 2 shows the effect of different reaction times on the morphology of CSs at a carbonization temperature of 270 °C and a reaction concentration of 0.06 g ml^−1^. Figure 2 shows that when the reaction time was 3 h, no distinct spherical shape is formed. Instead, irregularly shaped clusters formed. It can be inferred that hydrolysis may have just begun for EHL; therefore, more complete CSs have not yet been formed.

#### Effect of reaction concentration on the morphology of CSs

**Figure 3:**
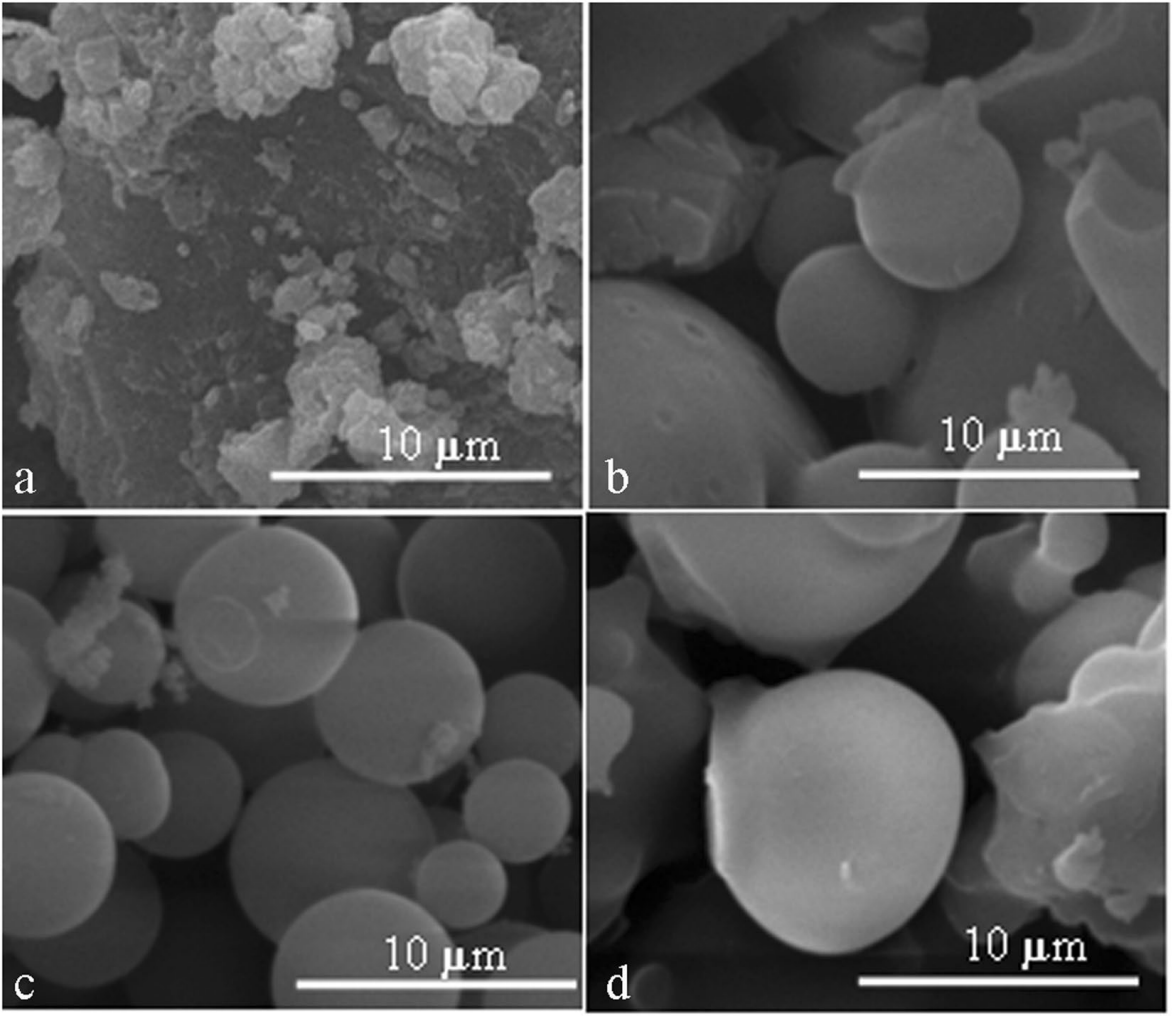
SEM images of extracted EHL (**a**) and carbon spheres at EHL concentrations of 0.04 g ml^−1^ (**b**), 0.06 g ml^−1^ (**c**) and 0.08 g ml^−1^ (**d**) (the carbonization temperature and time were 270 °C and 7 h, respectively).

The concentration of the EHL solution affects the formation of monodisperse carbon spheres, as the CSs are attracted to each other by van der Waals forces, leading to agglomerated collections of CSs^30^. Figure 3 depicts the SEM images of extracted EHL and carbon spheres formed at concentrations of 0.04 g ml^−1^, 0.06 g ml^−1^ and 0.08 g ml^−1^. The carbonization temperature and time were 270 °C and 7 h, respectively. With an increase in the solution concentration from 0.04 g ml^−1^ to 0.06 g ml^−1^, spherical particles gradually formed, ultimately forming separate particles with uniform dimensions/shapes. However, when the concentration was raised from 0.06 g ml^−1^ to 0.08 g ml^−1^, a cluster of agglomerated spheres formed. Therefore, the concentration of 0.06 g ml^−1^ is the best concentration for synthesis under the carbonization conditions applied in this study.

### The effects of microwave heating on CSs

#### Yield and proximate analysis

Hydrothermal carbonization on EHL resulted in carbon sphere and activated carbon sphere yields of 65.2% and 57.5%, respectively (Table 1). The CS yield was lower than the hydrothermal carbonization carbon yield. Heating enabled KOH to react more intensely with carbon, releasing more of its volatile content and hence lowering the carbon yield^31^.

**Table 1:** Yield and elementary analysis of EHL, carbon spheres (CSs) and activated carbon spheres (ACSs).

Samples	Yield (%)	C (%)	O (%)	N (%)	H (%)
EHL-raw	—	77.5	20.6	0.25	1.65
Hydrothermal Carbonization (CSs)	65.2	88.1	10.4	0.2	1.5
After microwave heating (ACSs)	57.5	95.2	3.5	0.1	1.2

The proportionate weight changes of four elements (C, N, H, and O) were measured after the activation process. The results showed that the concentrations of all four elements were drastically reduced. These results indicated that activation effectively removed impurities and improved the relative carbon proportion in the solid phase.

#### BET surface area and pore size distribution

**Figure 4:**
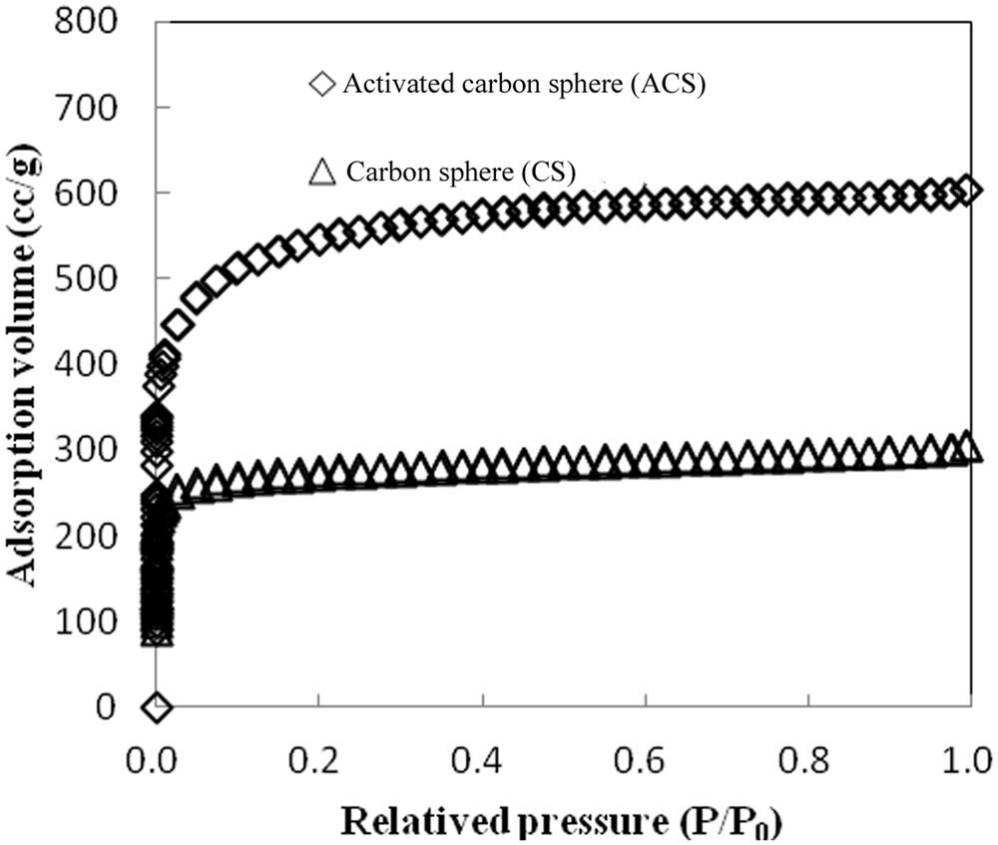
N_2_ adsorption isotherms at 77 K for CSs and KOH ACSs formed via microwave heating.

It is important to characterize the pore structure of carbon sphere materials. This characterization is most frequently conducted at cryogenic temperatures, namely, by the adsorption of an inert gas, such as N_2_ at its boiling point of 77 K under one atmosphere of pressure. Figure 4 displays the typical N_2_ adsorption/desorption isotherms at 77 K of the carbon spheres (carbonization temperature of 270 °C, carbonization time of 7 h, and concentration of 0.06 g ml^−1^) and KOH activated carbon spheres via microwave heating. A short duration of microwave heating of the KOH/CS mixture had a positive effect on the nitrogen adsorption capacity, which increased from 28 to 59 cc g^−1^, indicating that activation by KOH develops extensive porosity within the carbon spheres. These observations are confirmed by the pore structure parameters in Table 2. The values for BET specific surface area, mesopore surface area, micropore pore volume, and total pore volume were 1240 m^2^ g^−1^, 0.503 cc g^−1^ and 0.621 cc g^−1^, respectively. Both isotherm curves initially rose rapidly at very low relative pressures and then suddenly stopped rising; this behavior is attributed to the closing of adjacent pore walls such that multilayers of the activated carbon spheres could not be produced. The three isotherms were observed to be type I according to the Brunauer, Deming, Deming and Teller (BDDT) classification^32^.

**Table 2:** Characterization of carbon spheres and activated carbon spheres formed at a carbonization temperature of 270 °C, a carbonization time of 7 h, and a concentration of 0.06 g/ml.

Index	Carbon spheres	Activated carbon spheres
Surface area (m^2^/g)	384	1240
Micropore surface area (m^2^/g)	332	988
Micropore volume (cc/g)	0.066	0.503
Total pore volume (cc/g)	0.091	0.621

**Figure 5:**
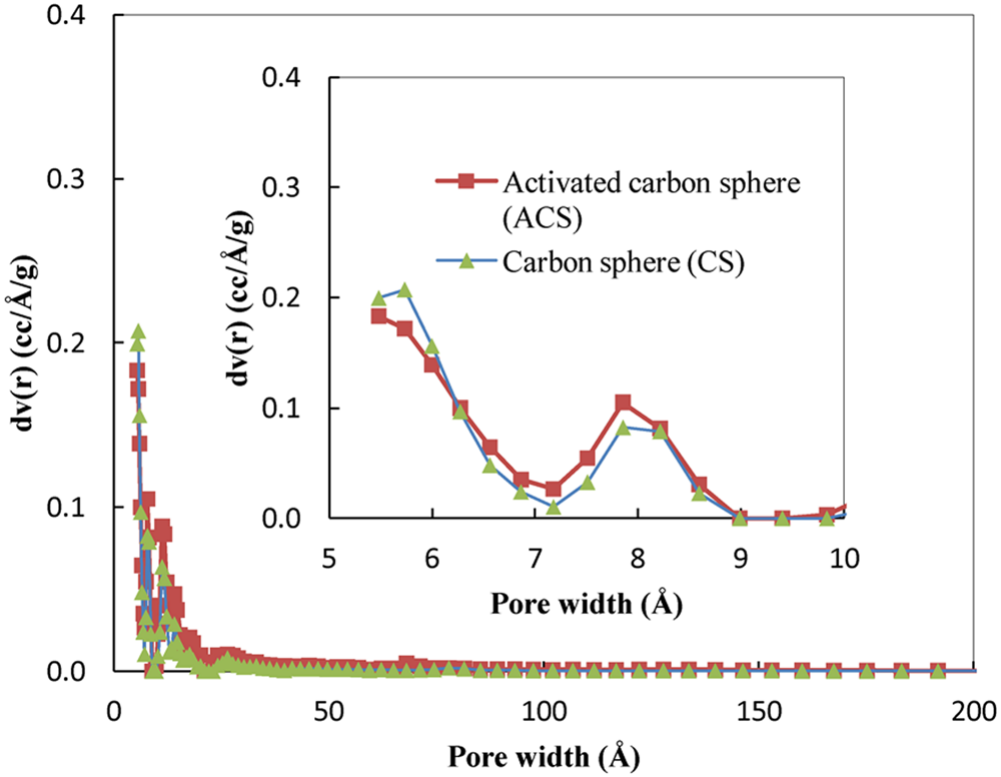
Pore size distribution of CSs and KOH ACSs formed via microwave heating.

Figure 5 illustrates the pore size distributions of CSs and KOH activated carbon spheres (ACSs) formed via microwave heating. The sharpest peak in the distributions occurred at pore diameters of 5 Å to 10 Å, which indicates that the majority of the pores fell within the micropore range. Carbon spheres activated by treatment with KOH and microwave heating may develop micropores (pore size < 2 nm) that enhance the adsorptive capacity for small molecules such as those of gases, according to the theory of the volume filling of micropores^32^.

The porosity and specific surface area of carbon spheres was characterized by N_2_ adsorption and desorption measurements at −196 °C. Table 3 summarizes the results obtained for carbon spheres formed at a carbonization temperature of 270 °C, a carbonization time of 7 h, and a concentration of 0.06 g ml^−1^. As expected, the HTC product showed a very low N_2_ adsorption capacity and a small BET surface area of 384 m^2^ g^−1^ as a result of pyrolysis at 270 °C. This BET surface area is comparable to that of carbon spheres derived from hemp stem hemicellulose at 600 °C (248 m^2^ g^−1^)^33^. After microwave heating, the BET surface area of ACSs increased from 248 m^2^ g^−1^ to 1278 m^2^ g^−1^, which occurred more efficiently (10 min) than the increase to 1276 m^2^ g^−1^ for hemicellulose-based ACSs with a KOH/ratio of 1.0 at 800 °C for 3.5 h^34^.

**Table 3:** Characteristic peaks of EHL and their assignments.

Wave number/cm^−1^	Characteristic peaks of functional groups and their assignments
1700	Non-conjugated carbonyl
1650–1655	Conjugated carbonyl
1600–1605, 1505–1515	Vibration of aromatic skeleton
1460–1470	C-H deformation vibration of methyl or methylene
1424	C-H plane deformation vibration on aromatic nucleus
1328	Syringyl
1266–1270	Guaiaretic nuclear methoxy C-O vibration
1220–1230	C-O vibration of the aromatic nucleus related to syringyl
1167	C-O-C stretching vibration in ester bond
1125–1130	Aromatic nucleus C-H vibration
1120	In-plane bending vibration of syringyl ring C-H
1030–1036	In-plane deformation vibration of aromatic C-H
833–835	Out-of-plane bending vibration of aromatic nucleus C-H

### FTIR analysis results

**Figure 6:**
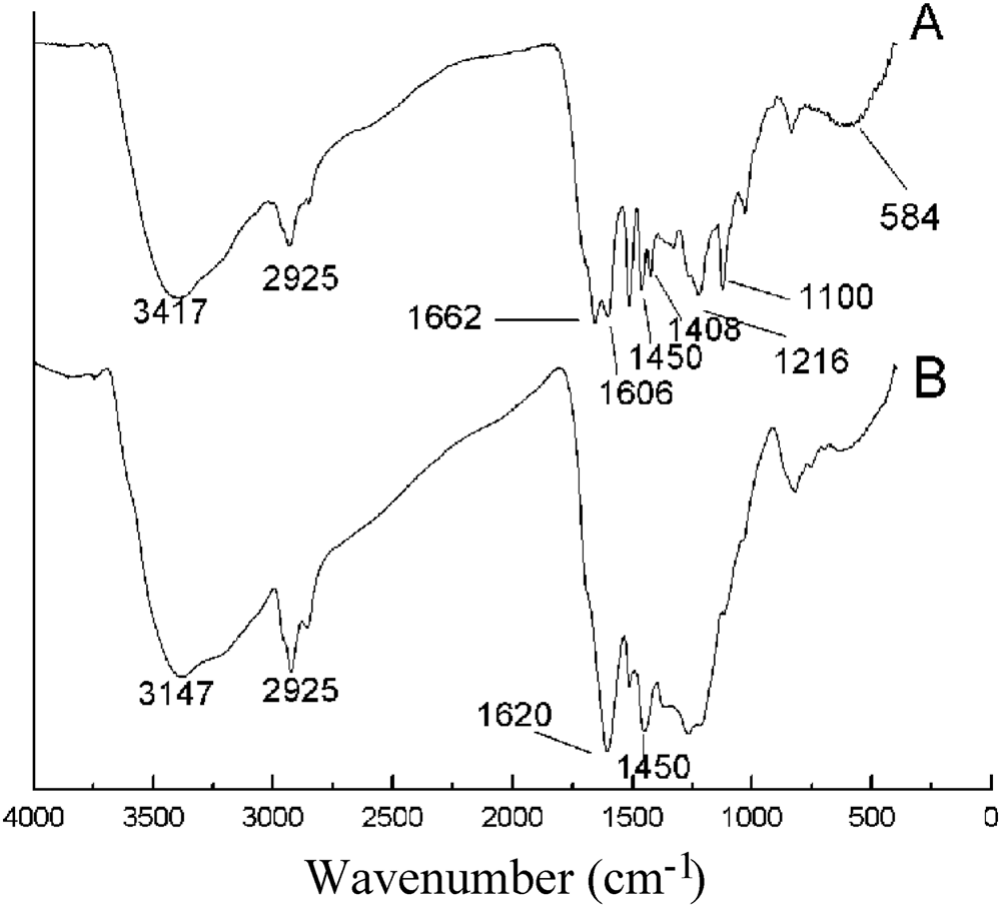
Infrared spectra of carbon spheres prepared by hydrothermal carbonization at a carbonization temperature of 270 °C, (**a**) carbonization time of 7 h, and a concentration of 0.06 g ml^−1^ (**A** is the infrared spectrogram of EHL; **B** is the infrared spectrogram of CS).

Figure 6 is an infrared spectrogram of the HTC products extracted from EHL at 270 °C for a reaction time of 7 h at a concentration of 0.06 g ml^−1^. In Fig. 6, Curve A corresponds to EHL before carbonization and Curve B corresponds to the product of HTC. Curve A shows a wide and strong peak at 3147 cm^−1^, which is the stretching vibration peak of the O-H bond, and the peak at 2925 cm^−1^ is the stretching vibration peak of C-H. The characteristic absorption of functional groups of EHL is mainly concentrated in the 800–1800 cm^−1^ range, where the peak at 1662 cm^−1^ is the deformation vibration peak of C_6_H_5_-C=O and that at 1606 cm^−1^ is the stretching vibration of the phenyl ring skeleton C=C; a strong stretching vibration peak of C=O occurs at 1450 cm^−1^, and the peak at 1000–1440 cm^−1^ corresponds to the stretching vibration peak of C-O bonds from the saccharide rings^25,35^.

Curve B suggests the presents of a large number of oxygen-containing functional groups (C=O and O-H) on the surface of the CSs. The peaks at 1620 cm^−1^ and 875–750 cm^−1^ correspond to the mixed stretching vibrations of C=O in conjugation with a C=C network in carbonaceous materials and out-of-plane C-H vibrations, respectively, which indicate the presence of aromatic nuclei on the CSs^36^. This result is coincident with a previous work by Smith and Chugtai^36^. Moreover, the presences of an aliphatic structure can be deduced from the stretching vibration peak of C-H at 2925 cm^−1^. Compared with Curve A for the raw material, in Curve B, the peak intensities at 1100 cm^−1^ and 3000–3700 cm^−1^ decrease, indicating the dehydration of EHL. Therefore, a comparison of Curves A and B shows that dehydration and aromatization occurred during the HTC of EHL, and many oxygen-containing functional groups such as C=O and C-O were present on the surface of the synthesized CSs^37,38^.

## Conclusion

The results of this study show that enzymatic hydrolysis lignins are potential precursors for the HTC preparation of high-quality activated carbon spheres. Microwave heating and KOH impregnation introduced pores into the activated carbon materials after a short heating period.

The prepared samples and materials were characterized according to different properties of the synthesized CSs, and the main conclusions are as follows: The results showed that when the reaction temperature was below 230 °C, spherical structures did not appear. When the temperature was above 230 °C, with the increase in temperature, the spherical structures became more uniform and showed better dispersion. Therefore, the reaction temperature mainly affects the morphology and dispersion of CSs. Within a certain range, a longer reaction time resulted in larger particle size of the CSs. At a reaction time was 7 h, the CSs reached their maximum particle size, i.e., 3–6 μm. Therefore, the reaction time is significantly affects the morphology and size of CSs. Hence, the optimized carbonization conditions are a temperature of 270 °C, a reaction time of 7 h and a concentration of 0.06 g ml^−1^. In addition, under the condition it can form the separate and uniform dimensions/shapes (3–6 μm). KOH activation and microwave heating were able to yield activated carbon spheres with favorable microporosity and adsorption properties with a BET surface area of 1278 m^2^ g^−1^. The surface area, micropore volume, mesopore volume, and surface oxygen content all increased after activation. Hemicellulose-derived carbon spheres exhibit excellent adsorption performance due to their abundant micropores and oxygen functionalities.

## Materials and Methods

### Materials

Raw EHL was obtained from Nanjing Chemical Reagent Co., Ltd. A 3% sodium hydroxide solution was obtained by mixing sodium hydroxide powder (Nanjing Chemical Reagent Co., Ltd) and distilled water according to the mass ratio, and a 2% dilute sulfuric acid solution (Nanjing Chemical Reagent Co., Ltd) was obtained by mixing 98% sulfuric acid (Nanjing Chemical Reagent Co., Ltd) and distilled water according to the volume ratio. All measurement instruments in this experiment were used at room temperature, with the temperature of the magnetic heated stirrer held at approximately 80 °C, and the temperature of the constant-temperature drying oven held at 60 °C to prevent the formation of mildew caused by the water content in the purified EHL; moreover, the temperature of the high-temperature box-type electric furnace was set to range from 190 to 270 °C.

### Extraction of EHL

In this study, EHL was extracted by the alkaline aqueous solution extraction method in which 100 g of unpurified EHL powder was weighed, placed in a 3 L conical flask, and combined with 1 kg of a 3% sodium hydroxide solution (sodium hydroxide powder mixed with distilled water according to the mass ratio). Then, a glass rod was used to stir the mixture and ensure that the powder was fully dissolved. The sample was left on a magnetic heated stirrer to react for 1 h at 65 °C. Then, the impurities were removed by vacuum filtration while the solution was still warm. The filtrate was poured into a beaker, and a 2% dilute sulfuric acid solution (98% sulfuric acid mixed with distilled water according to the volume ratio) was added. The filtrate was stirred while the dilute sulfuric acid solution was being added to adjust the pH value to 3. The solution was kept in a water bath for heat insulation and agglutination at 70 °C for 30 min. When granular lignin floated to the top of the solution, the lignin was sunk by stirring, and the solution was left to stand overnight. The upper clear layer of the solution was removed; the lower layer containing the target substance was separated using a centrifuge and then washed 4 times. The obtained EHL was placed in a constant-temperature drying oven at 60 °C. After the water content had largely evaporated, the sample was again transferred into the vacuum drying oven to fully evaporate the water.

### Synthesis, yield and elemental analysis of EHL-based carbon spheres

Two grams of the extracted EHL was weighed for use as a raw material, and it was placed in 50 ml of deionized water. Then, the solution was placed on a magnetic heated stirrer and stirred for 30 min to evenly disperse the EHL. The sample was then transferred to a 20 ml hydrothermal reactor lined with polytetrafluoroethylene at a filling rate of 50%. The sealed hydrothermal reactor was placed in a muffle furnace, and the temperature was set to 220, 230, 250, 270 or 280 °C for reaction times of 3, 5, or 7 h. The solution was naturally cooled to room temperature in the muffle furnace. The substances in the lower layer of the hydrothermal reactor were separated into a centrifuge tube, and a suitable amount of anhydrous ethanol was added. Then, the centrifuge tube was placed in a centrifuge and spun for 5 min before being removed. The carbonized product was dried and deemed ready for use.

The yields of the recovered solid products, referred to as CSs, were calculated using the following equation:

1 $$CS(wt \% )=\frac{{W}_{2}}{{W}_{1}}\times 100 \% $$

where CS is the yield of carbon spheres (wt%); *W*_*2*_ is the weight of the initial amount of the extracted EHL (g); and *W*_*1*_ is the amount of solid recovered.

2 $$ACS(wt \% )=\frac{{W}_{2}}{{W}_{1}}\times 100 \% $$

Here, CS is the yield of carbon spheres (wt%); *W*_*2*_ is the weight of the initial amount of the extracted EHL (g); and *W*_*1*_ is the amount of ACSs.

The bulk composition of the raw and activated carbon materials was analyzed for carbon, hydrogen, nitrogen and sulfur using a CHNS Elemental Analyzer (Elementar Analysensysteme, Vario MICRO).

### Microwave activation of the carbon spheres

KOH chemical activation was employed to develop micropores in the EHL-derived carbon spheres. The carbon spheres produced were impregnated with KOH at a KOH/char ratio of 1.0. The mixtures were stirred for 2 h, dried at 110 ± 5 °C overnight and stored in a desiccator.

For microwave activation, 30 g of KOH-impregnated char was added to a ceramic crucible in a cylindrical quartz reactor. The crucible was then placed in a 2450 MHz microwave applicator (Goldstar Co., Ltd.) with a nominal power output of 700 W and irradiated for 10 min under a 0.5 L/min flow of N_2_ humidified by bubbling through water^28^.

### Characterization of the carbon spheres

The samples were observed in a high-vacuum environment using a Model Quanta 200 scanning electron microscope manufactured by the U.S.-based FEI Company. Carbon spheres were dispersed in an anhydrous ethanol solution to prevent aggregation or crosslinking of the CSs in the solution. After ultrasonic oscillation for 15 min, the upper turbid anhydrous ethanol solution containing the sample was siphoned, dripped onto a clean glass slide and dried naturally. Care was taken to prevent dust from interfering with the drying process. To avoid interference with the electron beam caused by repulsive forces due to charge accumulation on the sample surface, the sample surface was sprayed with metal using an ion-sputtering apparatus before observation. During observation, an electron spectrometer was used to characterize the chemical elements contained in the sample.

The Brunauer-Emmett-Teller (BET) surface areas of the activated carbon samples were measured using N_2_ adsorption isotherms at 77 K with an automated gas sorption analyzer (Autosorb iQ_2_ MP, Quantachrome Instruments). The multipoint BET equations were used to calculate the BET surface areas using adsorption data recorded over the relative pressure range of 0.01 to 0.07. The total pore volume was derived from the amount of N_2_ adsorbed at a relative pressure of 0.99^39^, assuming that all of the pores were filled with liquid N_2_. Micropore volumes and surface areas were determined using the t-plot method.

The infrared spectral analyzer used in this study was a VERTEX-80v Fourier Transform Infrared Spectrometer. After the test sample was pressed into a KBr pellet, the infrared spectrum of the CSs was measured. The scanning range was 500–4000 cm^−1^.
